# Supplementary material for: Abiotic formation of alkylsulfonic acids in interstellar analog ices and implications for their detection on Ryugu
Source: Nat Commun. 2024 May 23;15:4409. doi: 10.1038/s41467-024-48684-5 (PMC11526038; doi:10.1038/s41467-024-48684-5)
Supplement: Supplementary file 1 — Supplementary Information [file 41467_2024_48684_MOESM1_ESM.pdf]

Supplementary Information

# Abiotic Formation of Alkylsulfonic Acids in Interstellar Analog Ices and Implications for their Detection on Ryugu

Mason McAnally[1,2], Jana Bocková[3], Ashanie Herath[1,2], Andrew M. Turner[1,2], Cornelia Meinert[3]*, Ralf I. Kaiser[1,2]*

[1]Department of Chemistry, University of Hawaii at Mānoa, Honolulu, HI 96822

[2]W.M. Keck Laboratory in Astrochemistry, University of Hawaii at Mānoa, Honolulu, HI 96822

[3]Université Côte d'Azur, Institut de Chimie de Nice, UMR 7272 CNRS, 06108 Nice, France

*Corresponding authors:

Cornelia.MEINERT@univ-cotedazur.fr, ralfk@hawaii.edu.

**Supplementary Note 1**

Two additional absorptions were assigned to carbon disulfide ($CS_2$), i.e., the asymmetric stretch ($\nu_3$) at 1520 $cm^{-1}$ and the $\nu_1 + \nu_3$ combination mode at 2166 $cm^{-1}$ [1]. The presence of carbonyl sulfide (OCS) is supported by the fundamental $\nu_1$ at 2042 $cm^{-1}$ [1]. Finally, ethane ($C_2H_6$) was assigned via the $\nu_5$ fundamental at 2882 $cm^{-1}$, the $\nu_{10}$ mode at 2977 $cm^{-1}$, the $\nu_{11}$ at 1465 $cm^{-1}$, and the $\nu_8 + \nu_{11}$ combination band at 2941 $cm^{-1}$ [2-4].

Isotopic shifting allowed for confirmation of small molecules, e.g., $\nu_3$ of $CS_2$ was observed at 1521 $cm^{-1}$ in the non-labeled experiment, while a new peak at 1460 $cm^{-1}$ appears in the $SO_2/^{13}CH_4$ ice consistent with $^{13}CS_2$[1]. Consequently, since $CS_2$ contains no hydrogen, no shifting is detected in the $SO_2/CD_4$ experiment for $CS_2$.

**Supplementary Note 2**

Changes in the infrared spectra during irradiation were kinetically fit to provide insight into formation mechanisms initiated by energetic electrons[5]. The column densities (molecules $cm^{-2}$) of the reactants and products were determined utilizing a modified Beer-Lambert equation[6] and the corresponding integrated absorption coefficients (Supplemntary Table 12) during the irradiation (Fig. 5). The changes in column density of each reactant and product are summarized in Supplemntary Table 13 and the kinetic scheme utilized in these fits are cataloged in Supplemntary Table 14 as well as rate constants for each reaction in each dose.

A set of coupled differential equations were solved numerically under the assumption that the reactions followed elementary first and second-order growth and decay profiles. By combining first-order decay and second-order decay reactions, the column density of $SO_2$ was fit during irradiation.

The column density of sulfur dioxide ($\nu_3$, 1330 $cm^{-1}$) declines during irradiation, with the high dose experiment showing an 85% reduction in column density ($-1.6 \times 10^{17}$ molecules $cm^{-2}$)—over five times larger than the low dose experiment ($-2.9 \times 10^{16}$ molecules $cm^{-2}$). The 1000 nA and 5000 nA experiments also included the reformation of $SO_2$ via radical-radical recombination of SO and atomic oxygen. The rate constant of the decomposition of $SO_2$ to SO and atomic oxygen is markedly higher in the 5000 nA experiment ($5.3 \times 10^{-5}$ $s^{-1}$) compared to the 100 nA experiment

($5.7 \times 10^{-6}$ $s^{-1}$). The reduction in column density of methane—tracked by $\nu_2$ at 1300 $cm^{-1}$—during the irradiation occurs in all three dose experiments, and the most substantial change appears in the high dose experiment where the column density changes from $1.9 \times 10^{17}$ molecules $cm^{-2}$ to $4.3 \times 10^{16}$ molecules $cm^{-2}$. Utilizing a similar scheme to $SO_2$, the decay of methane was fit along with an additional second-order decay to form unknown products in the 5000 nA experiment. This decrease in column density of methane coincides with an increase in the column density of the methyl radical ($\nu_3$, 3160 $cm^{-1}$) in the low dose experiment and the production of ethane ($\nu_{10}$, 2976 $cm^{-1}$) in the low, medium, and high dose experiments. The methyl radical is consumed predominately by radical-radical recombination to form ethane. The 5000 nA experiment shows the highest rate of ethane production of $2.8 \times 10^{-17}$ $cm^2$ molecules$^{-1}$ $s^{-1}$.

Since the 100 nA experiment provides identification of the methyl radical, the kinetic scheme was first fit utilizing these data. The formation of known intermediates such as $SO_3$ and the methyl radical reveals a possible pathway towards forming alkylsulfonic acids (ASAs). Sulfur trioxide—contains two second-order growths: the addition of atomic oxygen to $SO_2$ and a biomolecular reaction of $SO_2$ producing SO and $SO_3$. Two decay reactions are derived from the kinetic scheme. The first is a first-order decay towards unknown products. In the second, $SO_3$ reacts with the methyl radical to form $CH_3SO_3$. This reaction shows an order of magnitude increase between the 100 nA and 1000 nA experiments and between the 1000 nA and 5000 nA experiments. Previous computational work has described this reaction (Supplemntary Figure 8), where $CH_3SO_3$ initially goes through a van der Waals complex before a 1 kJ $mol^{-1}$ barrier transition state[7]. Matrix effects and suprathermal reactants may allow the crossing of this minor barrier. After forming $CH_3SO_3$, hydrogen addition produces methylsulfonic acid. Since the band strengths of ASAs are unknown, the fits were completed by estimating the S=O stretching strength which was $5.0 \times 10^{-17}$ cm molecule$^{-1}$ before normalizing the fit to experimental data. This kinetic scheme accurately predicts the delay in the production of methylsulfonic acid (MSA) in the 100 nA experiment and the more rapid productions observed in the 1000 nA and 5000 nA experiments. Additionally, the rates derived for hydrogen addition to $CH_3SO_3$ show an increase from $1.2 \times 10^{-19}$ $cm^2$ molecules$^{-1}$ $s^{-1}$ in the 100 nA experiment to $9.0 \times 10^{-18}$ $cm^2$ molecules$^{-1}$ $s^{-1}$, i.e., a 75 times increase in reaction rate.

This kinetic scheme represents one of the first attempts at understanding the production of organosulfur compounds in interstellar analog ices. This scheme provides insight into possible formation mechanisms of ASAs in $SO_2/CH_4$ ices and produces reaction rates applicable to interstellar icy mantles. Here, the synthesis of methylsulfonic acid occurs through methyl radical addition to sulfur trioxide before hydrogen addition to form MSA.

**Supplementary Note 3**

Changes in the infrared spectra collected during TPD (Supplemntary Figure 7) can reveal the sublimation temperature of compounds as well as thermal reactions. The TPD profiles indicate a decrease in column density starting at 30 K for methane and ethane, while $SO_2$ and $SO_3$ decrease at 110 K. The peak at 1200 cm$^{-1}$ assigned to S=O stretching associated with compounds such as ASAs shows a decrease in signal at 250 K during the TPD in the low and medium dose experiments. The high dose experiment shows two increases in intensity starting at 150 K and 210 K and two decreases in signal at 170 K and 300 K. The changes in intensity in the high dose experiment suggest thermal reactions may play a notable role in the chemistry of these sulfur-containing ices and could contribute to the formation of ASAs.

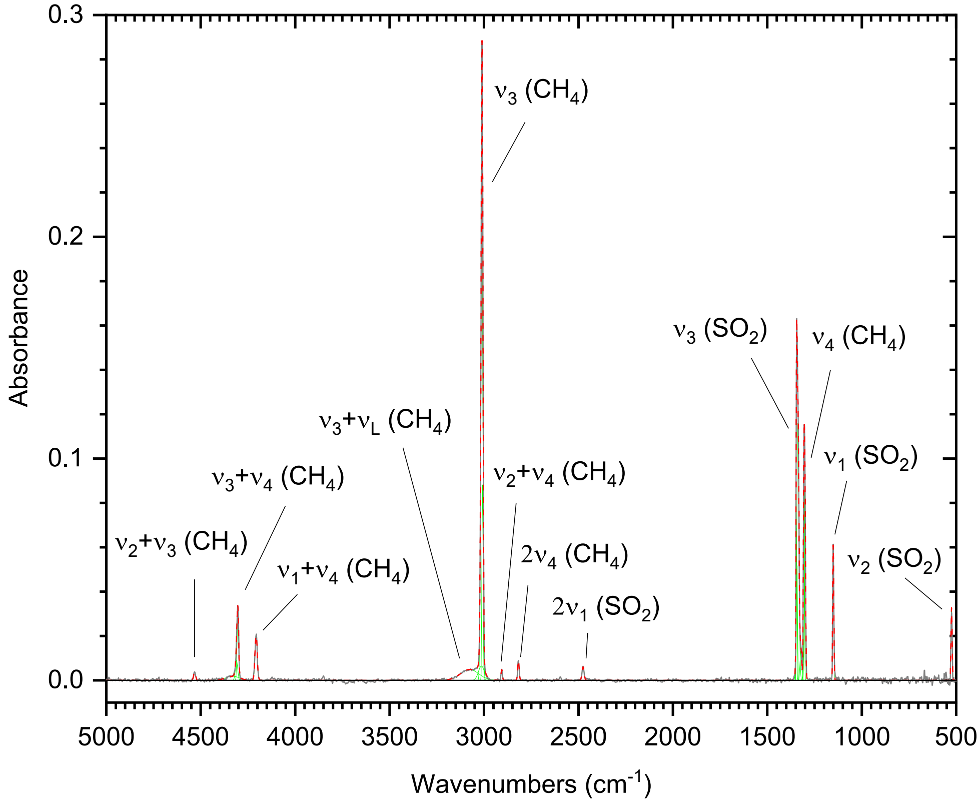


**Supplementary Figure 1.** Infrared spectra of $SO_2/CH_4$ ice at 10 K before irradiation. The original spectrum (gray) is deconvoluted showing peaks associated with reagents (green). These deconvolution peaks sum to the peak fitted spectrum (red dashed).

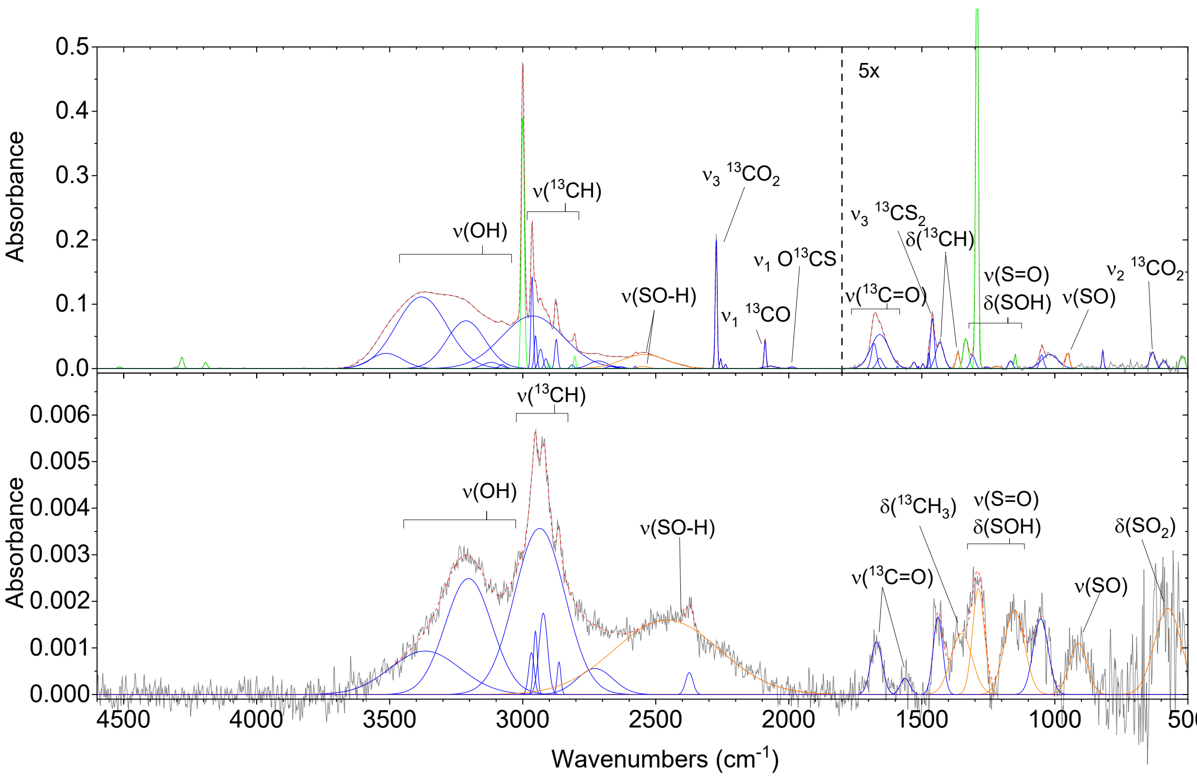


**Supplementary Figure 2.** Infrared spectra of $SO_2$/$^{13}CH_4$ ice at 10 K after irradiation (top) and at 320 K (bottom). The original spectrum (gray) is deconvoluted showing peaks associated with reagents (green), peaks associated with alkylsulfonic acids (orange) and other irradiation products (blue). These deconvolution peaks sum to the peak fitted spectrum (red dashed). Peak positions and assignments for after irradiation can be found in Supplementary Table 7 and for the residue in Supplementary Table 8.

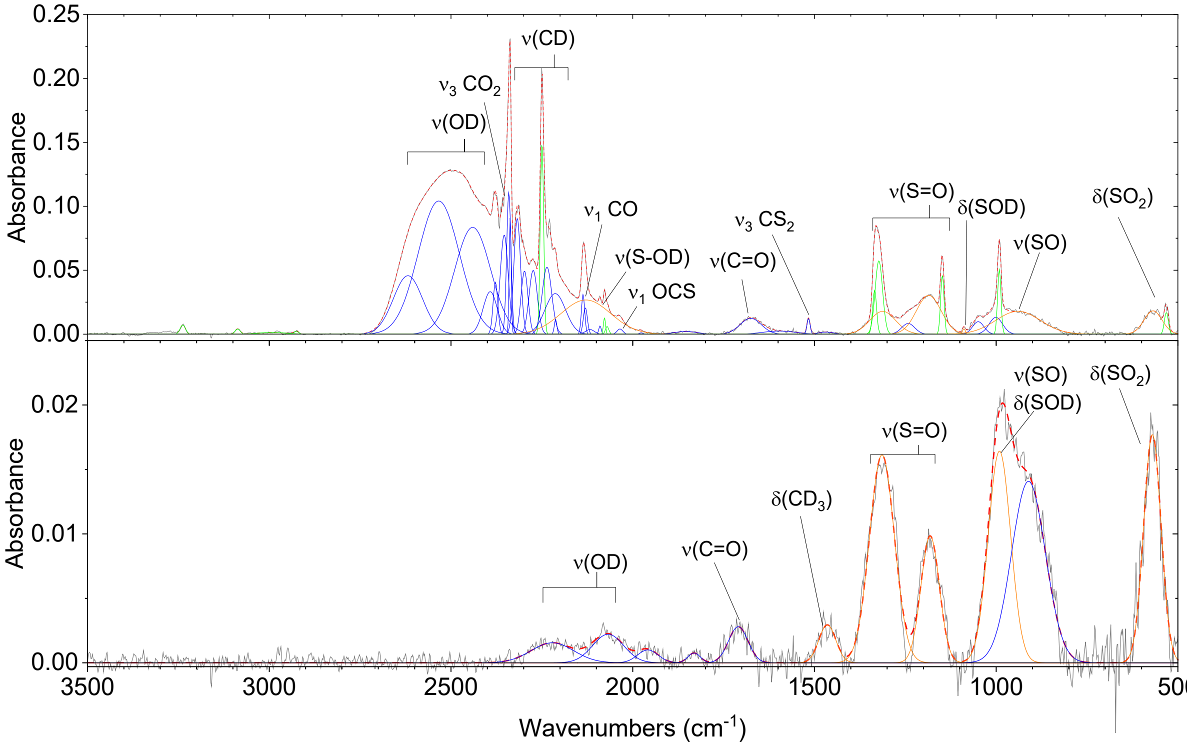


**Supplementary Figure 3.** Infrared spectra of $SO_2/CD_4$ ice at 10 K after irradiation (top) and at 320 K (bottom). The original spectrum (gray) is deconvoluted showing peaks associated with reagents (green), peaks associated with alkylsulfonic acids (orange) and other irradiation products (blue). These deconvolution peaks sum to the peak fitted spectrum (red dashed). Peak positions and assignments for after irradiation can be found in Supplementary Table 9 and for the residue in Supplementary Table 10.

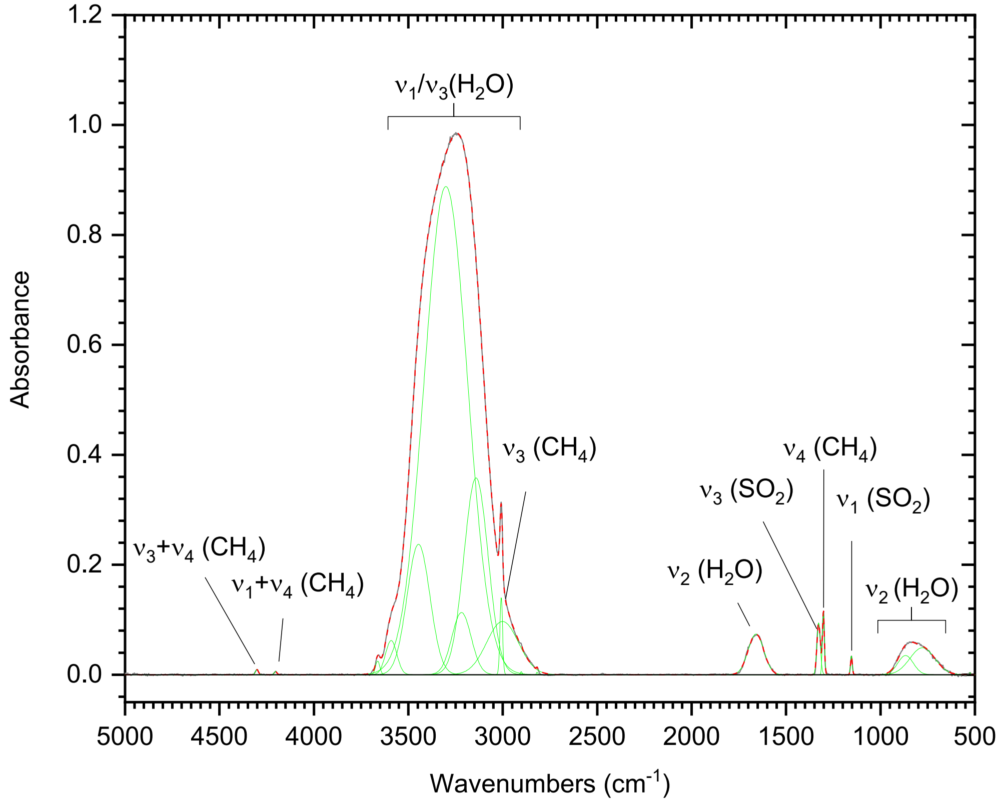


**Supplementary Figure 4.** Infrared spectra of $SO_2/H_2O/CH_4$ ice at 10 K before irradiation. The original spectrum (gray) is deconvoluted showing peaks associated with reagents (green). These deconvolution peaks sum to the peak fitted spectrum (red dashed).

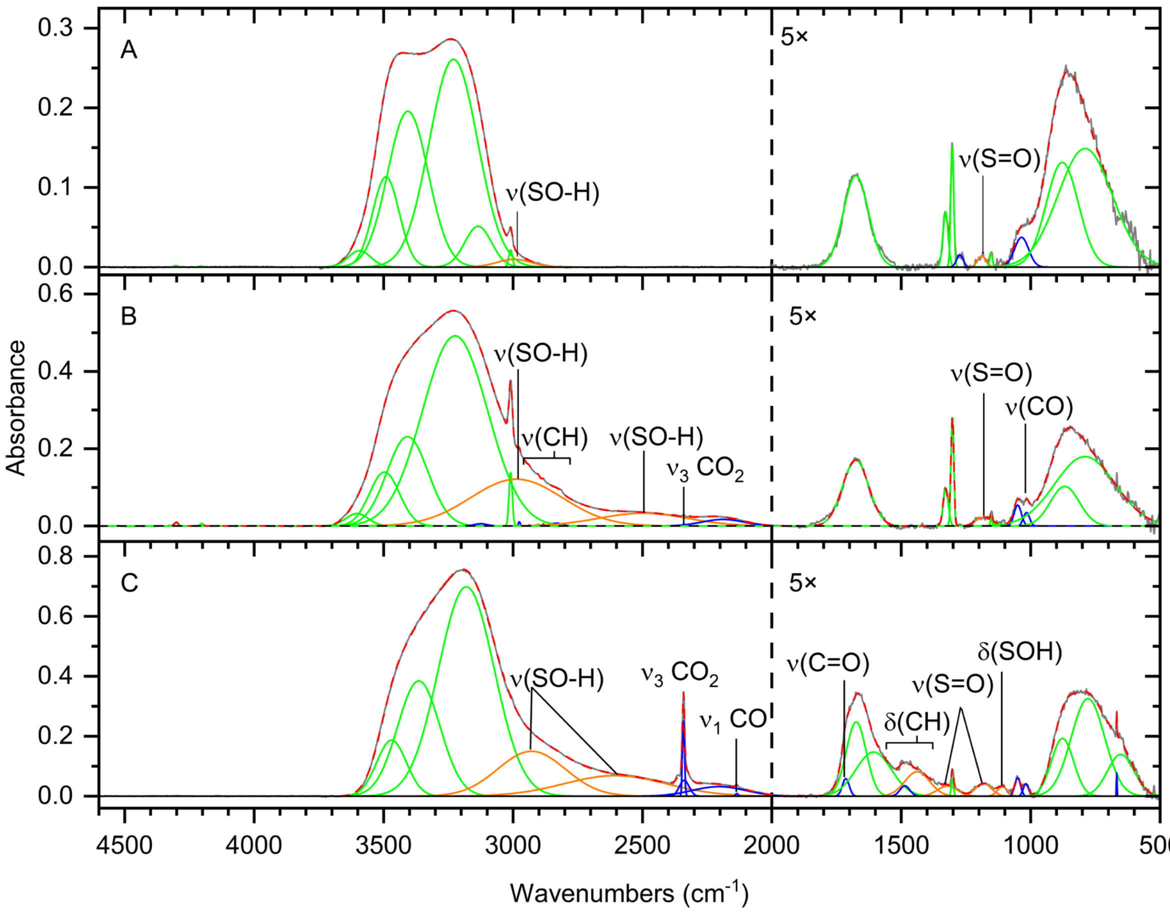


**Supplementary Figure 5.** FTIR spectra of $SO_2/H_2O/CH_4$ ices at 10 K after irradiation for 1 hour at (A) 100 nA, (B) 1000 nA, and (C) 5000 nA current. The original spectrum (gray) is deconvoluted showing peaks associated with reagents (green), functional groups associated with alkylsulfonic acids (orange), and new irradiation products (blue). These deconvolutions sum to the peak fitted spectrum (red dashed). The data to the right of the dashed vertical line at 2000 $cm^{-1}$ is magnified for clarity.

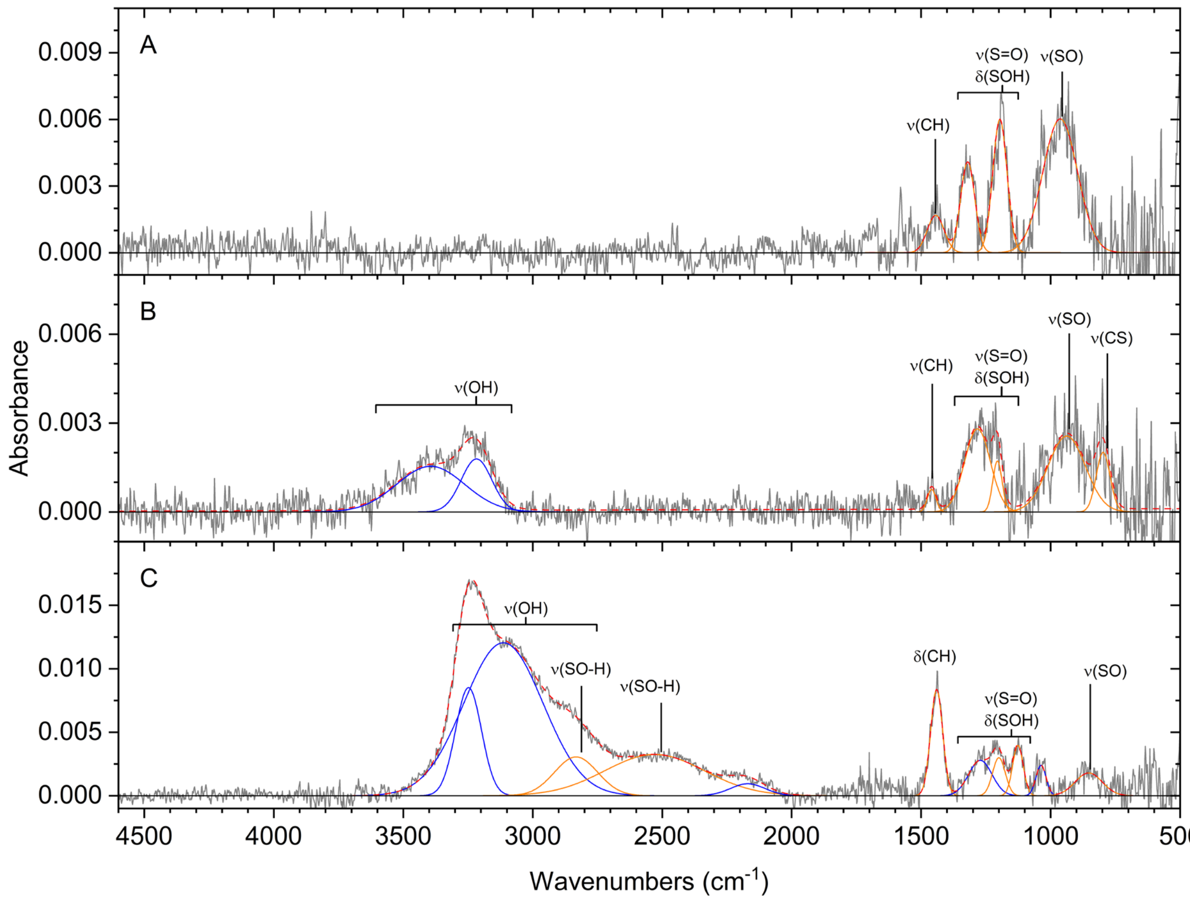


**Supplementary Figure 6.** FTIR spectra of $SO_2/H_2O/CH_4$ ices at 320 K after irradiation for 1 hour at (A) 100 nA, (B) 1000 nA, and (C) 5000 nA current. The original spectrum (gray) is deconvoluted showing functional groups associated with alkylsulfonic acids (orange), and new irradiation products (blue). These deconvolutions sum to the peak fitted spectrum (red dashed).

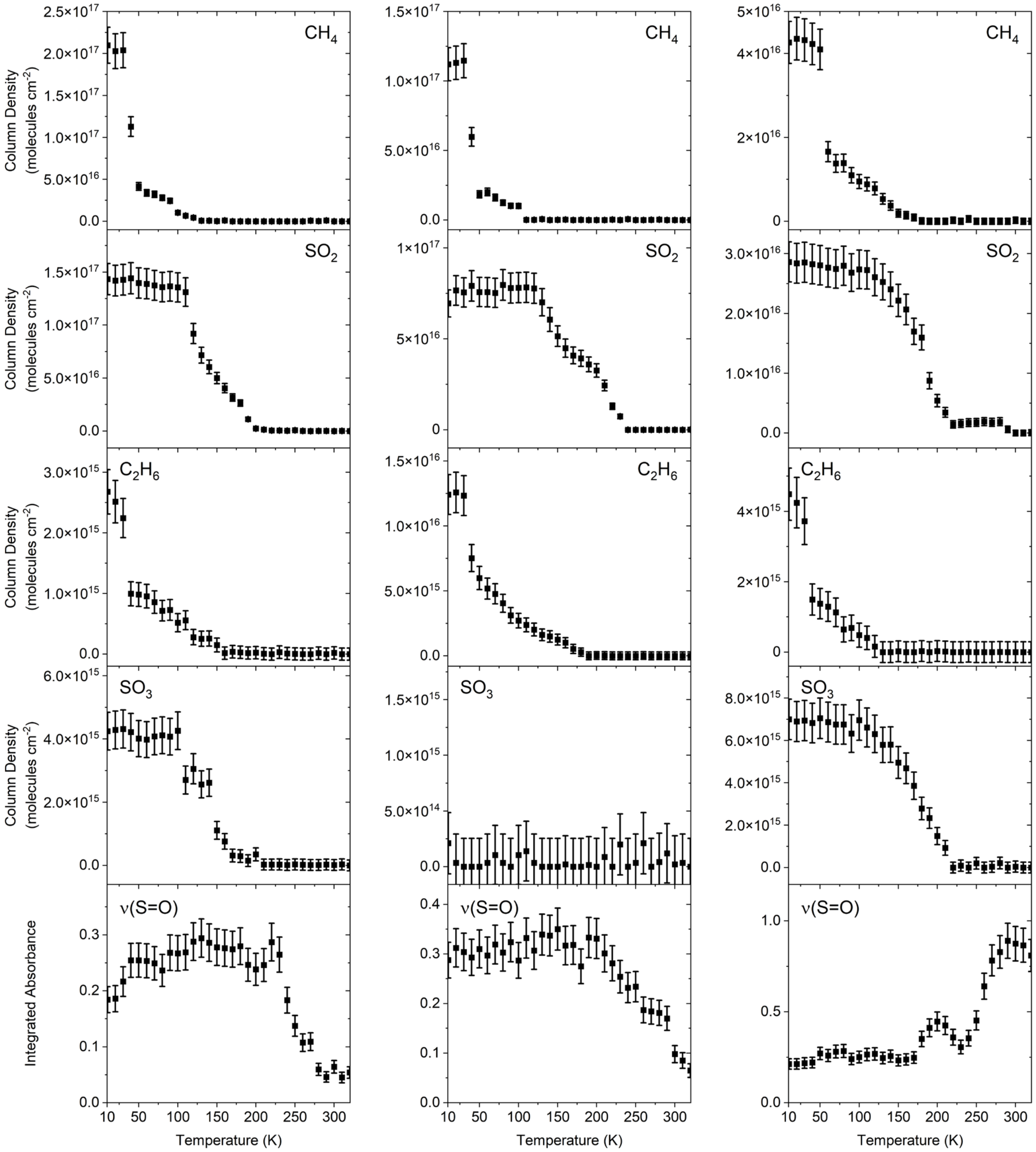


**Supplementary Figure 7. Temporal evolution of selected infrared peaks during TPD in the $SO_2$/$CH_4$ experiments.** The TPD profiles of selected infrared peaks of the low (left), medium (center), and high (right) dose. Methane, sulfur dioxide, ethane, sulfur trioxide, and *ν*(S=O) were tracked by the 1300 $cm^{-1}$, 1330 $cm^{-1}$, 2976 $cm^{-1}$, 1380 $cm^{-1}$, and 1200 $cm^{-1}$ peaks, respectively. Error bars are calculated from instrumental noise and error in ice thickness measurements.

**Supplementary Table 1.** Infrared absorption assignments for $SO_2/CH_4$ ices before irradiation.

| Peaks before irradiation (10 K) | | |
|---|---|---|
| Position ($cm^{-1}$) | Identity | References |
| 4533 | $\nu_2 + \nu_3$ $CH_4$ | 2 |
| 4332 | $\nu_3 + \nu_4$ $CH_4$ | 2 |
| 4311, 4303 | $\nu_3 + \nu_4$ $CH_4$ | 2 |
| 4207 | $\nu_1 + \nu_4$ $CH_4$ | 2 |
| 3077 | $\nu_3 + \nu_L$ $CH_4$ | 2 |
| 3014, 3012, 3008 | $\nu_3$ $CH_4$ | 2 |
| 2906 | $\nu_1$ $CH_4$ | 2 |
| 2818 | $\nu_2+\nu_4$ $CH_4$ | 2 |
| 2476 | $2\nu_1$ $SO_2$ | 8 |
| 1346, 1343, 1338, 1322 | $\nu_3$ $SO_2$ | 8 |
| 1306, 1301 | $\nu_4$ $CH_4$ | 2 |
| 1151 | $\nu_1$ $SO_2$ | 8 |
| 524 | $\nu_2$ $SO_2$ | 8 |

**Note.** "L" is used to define the lattice vibration

**Supplementary Table 2.** Infrared absorption assignments for $SO_2/CH_4$ experiments.

| Peaks after irradiation (10 K) | | | | | | | |
|---|---|---|---|---|---|---|---|
| Position ($cm^{-1}$) | Assignment | Dose (nA) | References | Position ($cm^{-1}$) | Assignment | Dose (nA) | References |
| 4533 | $\nu_2+\nu_3$ ($CH_4$) | 100 | 2 | 2115 | $\nu$(OH) | 1000 | 9 |
| 4306 | $\nu_3+\nu_4$ ($CH_4$) | 100, 1000, 5000 | 2 | 2087 | $\nu$(C≡C) | 5000 | 9 |
| 4206 | $\nu_1+\nu_4$ ($CH_4$) | 100, 1000, 5000 | 2 | 2037, 2042 | $\nu_1$ (OCS) | 1000, 5000 | 1 |
| 3849 | $\nu$(OH) | 100 | 9 | 1711, 1719 | $\nu$(C=O) | 100, 1000, 5000 | 9 |
| 3730, 3701 | $\nu_1+\nu_3$ ($CO_2$) | 100, 1000, 5000 | 10 | 1695, 1689 | $\nu$(C=O) | 1000, 5000 | 9 |
| 3514–3629 | $\nu$(OH) | 100, 1000, 5000 | 9 | 1668 | $\nu$(C=O) | 5000 | 9 |
| 3399–3456 | $\nu$(OH) | 100, 1000, 5000 | 9 | 1619 | $\nu$(C=O) | 100 | 9 |
| 3207–3251 | $\nu$(OH) | 100, 1000, 5000 | 9 | 1521 | $\nu_3$ ($CS_2$) | 5000 | 1 |
| 3100, 3092 | $\nu$(OH) | 1000, 5000 | 9 | 1496 | $\nu$(C=C), $\delta$(CH) | 1000, 5000 | 9 |
| 3049, 3069 | $\nu$(OH) | 100, 5000 | 9 | 1464, 1469 | $\delta$(CH), $\nu_{11}$ $C_2H_6$ | 1000, 5000 | 2,9 |
| 3025 | $\nu$(CH) | 1000 | 9 | 1434 | $\delta$(CH) | 5000 | 9 |
| 3008, 3010, 3018 | $\nu_3$ $CH_4$ | 100, 1000, 5000 | 2 | 1396, 1382 | $\nu_3$ ($SO_3$) | 100, 5000 | 1 |
| 2978 | $\nu_{10}$ ($C_2H_6$) | 100, 1000, 5000 | 2 | 1330 | $\nu_3$ ($SO_2$) | 100, 1000, 5000 | 8 |
| 2971 | $\nu$(OH) | 1000 | 9 | 1303 | $\nu_4$ ($CH_4$) | 100, 1000, 5000 | 2 |
| 2941 | $\nu_8+\nu_{11}$ ($C_2H_6$) | 100, 1000, 5000 | 2 | 1270 | $\nu$(S=O) | 5000 | 9 |
| 2931, 2934 | $\nu$(CH) | 100, 5000 | 9 | 1241 | $\nu$(S=O) SA | 1000 | 11 |
| 2905 | $\nu_1$ $CH_4$ | 100 | 2 | 1210 | $\nu$(S=O) ASA, SA | 1000, 5000 | 9,11-13 |
| 2899 | $\nu$(SO-H) ASA | 5000 | 9,12,13 | 1166, 1177 | $\nu$(S=O) | 100, 5000 | 9 |
| 2882 | $\nu_5$ ($C_2H_6$) | 100, 1000, 5000 | 2 | 1149 | $\nu_1$ $SO_2$ | 100, 1000, 5000 | 8 |
| 2825 | $\nu$(CH) | 5000 | 9 | 1119 | $\delta$(SOH) ASA, SA | 5000 | 9,11-13 |
| 2818 | $\nu_2+\nu_4$ ($CH_4$) | 100, 1000, 5000 | 2 | 1100, 1103 | $\nu$(S=O) | 5000 | 9 |
| 2743, 2699 | $\nu$(OH) | 1000, 5000 | 9 | 1046, 1050 | $\nu$(SO) | 1000, 5000 | 9 |
| 2523–2588 | $\nu$(SO-H) ASA | 1000, 5000 | 9,12,13 | 961, 996 | $\rho$(CH) ASA, $\nu$(SO) SA | 1000 | 9,11-13 |
| 2476 | $\nu$(C≡C) | 100 | 9 | 887, 876 | $\nu$(SO), $\rho$(CH) | 1000 | 9,11 |
| 2409, 2447, 2437 | $\nu$(OH) | 1000, 5000 | 9 | 724, 786 | $\nu$(CS) ASA | 1000, 5000 | 9,12,13 |
| 2367 | $\nu$(C≡C) | 1000, 5000 | 9 | 679 | OH Torsion | 5000 | 9 |
| 2335, 2347, 2342 | $\nu_3$ ($CO_2$) | 100, 1000, 5000 | 10 | 668 | $\nu_2$ ($CO_2$) | 100, 1000, 5000 | 10 |
| 2276 | $\nu$(C≡C) | 1000 | 9 | 653, 656 | $\delta$(OH) alcohols | 1000, 5000 | 9 |
| 2259, 2261 | $\nu$(OH) acid | 1000, 5000 | 9,11 | 577, 590 | $\delta$(SO) | 1000, 5000 | 9 |
| 2234, 2237 | $\nu$(C≡C) | 100, 5000 | 9 | 526 | $\nu_2$ ($SO_2$) | 100, 1000 | 8 |
| 2166 | $\nu_1+\nu_3$ ($CS_2$) | 5000 | 1 | | | | |
| 2138 | $\nu_1$ (CO) | 100, 1000, 5000 | 1 | | | | |

Note δ and ρ are used to describe the bending and rocking vibrational modes, respectively.

**Supplementary Table 3.** Infrared absorption assignments for $SO_2/CH_4$ experiments.

| Infrared absorption assignments for $SO_2/CH_4$ experiments | | | |
|---|---|---|---|
| Residue (320 K) | | | |
| Position ($cm^{-1}$) | Assignment | Dose (nA) | References |
| 3410 | *v*(OH) | 100 | 9 |
| 3213, 3233, 3246 | *v*(OH) | 100, 1000, 5000 | 9 |
| 3073, 3056 | *v*(OH) | 1000, 5000 | 9 |
| 2845 | *v*(SO-H) ASA | 5000 | 9,11 |
| 2426 | *v*(SO-H) ASA | 1000 | 9 |
| 2377 | *v*(C≡C) | 5000 | 9 |
| 2323 | *v*(C≡C) | 1000, 5000 | 9 |
| 1725, 1730 | *v*(C=O) | 100, 5000 | 9 |
| 1700, 1695 | *v*(C=O) | 1000, 5000 | 9 |
| 1618, 1613 | *v*(C=O) | 100, 5000 | 9 |
| 1520, 1510, 1511 | $CH_2$ scissoring, *v*(C=C) | 100, 1000, 5000 | 9 |
| 1416, 1440 | *δ*(CH) ASA | 1000, 5000 | 9,12,13 |
| 1297 | *v*(S=O) ASA | 1000, 5000 | 9,12,13 |
| 1268 | *δ*(OH) SA, *v*(S=O) ASA | 100, 5000 | 9 |
| 1186 | *v*(S=O) ASA, SA | 1000 | 9,11-13 |
| 1116 | *δ*(SOH) ASA, SA | 5000 | 9,11-13 |
| 1045 | *v*(CO) | 1000, 5000 | 9 |
| 1023 | *v*(CO) | 5000 | 9 |
| 909, 864 | *ρ*(CH) ASA, *v*(SO) SA | 100, 5000 | 9,11-13 |
| 771 | *v*(CS) ASA | 1000, 5000 | 9,12,13 |
| 647, 621 | δ(OH) alcohols | 100, 5000 | 9 |

**Supplementary Table 4.** Infrared absorption assignments for $SO_2/^{13}CH_4$.

| Peaks after irradiation (10 K) | | | | | |
|---|---|---|---|---|---|
| Position ($cm^{-1}$) | Assignment | References | Position ($cm^{-1}$) | Assignment | References |
| 4515 | $\nu_2 + \nu_3$ $^{13}CH_4$ | 2 | 1679 | $\nu(^{13}C{=}O)$ | 9 |
| 4281 | $\nu_3 + \nu_4$ $^{13}CH_4$ | 2 | 1659 | $\nu(^{13}C{=}O)$ | 9 |
| 4192 | $\nu_1 + \nu_4$ $^{13}CH_4$ | 2 | 1658 | $\nu(^{13}C{=}O)$ | 9 |
| 3513 | $\nu(OH)$ | 9 | 1592 | $\nu(^{13}C{=}O)$ | 9 |
| 3380 | $\nu(OH)$ | 9 | 1530 | $\nu(^{13}C{=}O)$ | 9 |
| 3214 | $\nu(OH)$ | 9 | 1513 | $\nu(^{13}C{=}O)$ | 9 |
| 3119 | $\nu(OH)$ | 9 | 1496 | $\nu(^{13}C{=}^{13}C)$, $\delta(^{13}CH)$ | 9 |
| 3078 | $\nu(OH)$ | 9 | 1474 | $\delta(^{13}CH)$ | 9 |
| 3000 | $\nu_3$ $^{13}CH_4$ | 2 | 1460 | $\nu_3$ $(^{13}CS_2)$ | 1 |
| 2965 | $\nu_{10}$ $^{13}C_2H_6$ | 2 | 1433 | $\delta(^{13}CH)$ | 9 |
| 2963 | $\nu(OH)$ | 9 | 1365 | $\delta(^{13}CH_3)$ ASA | 9,12,13 |
| 2952 | $\nu(^{13}CH)$ | 9 | 1335 | $\nu_3$ $SO_2$ | 8 |
| 2933 | $\nu_8 + \nu_{11}$ $^{13}C_2H_6$ | 2 | 1309 | $\nu(S{=}O)$ | 9 |
| 2913 | $\nu(^{13}CH)$ | 9 | 1292 | $\nu_4$ $^{13}CH_4$ | 2 |
| 2901 | $\nu_1$ $^{13}CH_4$ | 2 | 1255 | $\nu(S{=}O)$ SA, $\delta(SOH)$ | 11 |
| 2874 | $\nu_5$ $^{13}C_2H_6$ | 2 | 1217 | $\nu(S{=}O)$ ASA, SA | 9,11-13 |
| 2815 | $\nu(^{13}CH)$ | 9 | 1166 | $\nu(S{=}O)$ | 9 |
| 2805 | $\nu_2{+}\nu_4$ $^{13}CH_4$ | 2 | 1149 | $\nu_1$ $SO_2$ | 8 |
| 2716 | $\nu(OH)$ | 9 | 1050 | $\nu(SO)$ | 9 |
| 2659 | $\nu(^{13}CH)$ | 9 | 1019 | $\nu(^{13}CO)$ | 9 |
| 2631 | $\nu(^{13}CH)$ | 9 | 952 | $\rho(CH_3)$ ASA, $\nu(SO)$ | 9,11-13 |
| 2576 | $2\nu_4$ $^{13}CH_4$ | 2 | 820 | $\nu(SO)$, $\rho(CH)$ | 9 |
| 2547 | $\nu(SO\text{-}H)$ ASA | 9,12,13 | 633 | $\nu_2$ $^{13}CO_2$ | 10 |
| 2544 | $\nu(SO\text{-}H)$ ASA | 9,12,13 | 591 | $\delta(SO)$ ASA | 9,12,13 |
| 2273 | $\nu_3$ $^{13}CO_2$ | 10 | 520 | $\nu_2$ $SO_2$ | 8 |
| 2256 | $\nu_3$ $^{13}CO_2$ | 10 | | | |
| 2238 | $\nu(^{13}C{\equiv}^{13}C)$ | 9 | | | |
| 2089 | $\nu_1$ $(^{13}CO)$ | 10 | | | |
| 2070 | $\nu(^{13}C{\equiv}^{13}C)$ | 9 | | | |
| 1988 | $\nu_1$ $(O^{13}CS)$ | 1 | | | |

**Supplementary Table 5.** Infrared absorption assignments for $SO_2/^{13}CH_4$.

| Residue (320 K) | | |
|---|---|---|
| Position ($cm^{-1}$) | Assignment | References |
| 3367 | $\nu$(OH) | 9 |
| 3204 | $\nu$(OH) | 9 |
| 2967 | $\nu(^{13}CH)$ | 9 |
| 2952 | $\nu(^{13}CH)$ | 9 |
| 2936 | $\nu$(OH) | 9 |
| 2923 | $\nu(^{13}CH)$ | 9 |
| 2864 | $\nu(^{13}CH)$ | 9 |
| 2728 | $\nu$(OH) | 9 |
| 2458 | $\nu$(SO-H) ASA | 9,12,13 |
| 2374 | $\nu(^{13}C\equiv^{13}C)$ | 9 |
| 1671 | $\nu(^{13}C=O)$ | 9 |
| 1563 | $\nu(^{13}C=O)$ | 9 |
| 1439 | $\delta(^{13}CH_3)$ ASA | 9,12,13 |
| 1351 | $\delta(^{13}CH_3)$ ASA | 9,12,13 |
| 1287 | $\nu$(S=O) ASA, SA | 9,11-13 |
| 1152 | $\delta$(SOH) ASA, SA | 9,11-13 |
| 1053 | $\nu$(SO) | 9 |
| 912 | ν(S–OH) ASA, SA | 9,11-13 |
| 576 | $\delta(SO_2)$ ASA | 9,12,13 |

**Supplementary Table 6.** Infrared absorption assignments for $SO_2/CD_4$.

| Peaks after irradiation (10 K) | | | | | |
|---|---|---|---|---|---|
| Position ($cm^{-1}$) | Assignment | References | Position ($cm^{-1}$) | Assignment | References |
| 3238 | $\nu_3 + \nu_4$ $CD_4$ | 14 | 1468 | $\delta$(CD) | 9 |
| 3087 | $\nu_1 + \nu_4$ $CD_4$ | 14 | 1334 | $\nu_3$ $SO_2$ | 8 |
| 2992 | $\nu_1$ $CHD_3$ | 15 | 1323 | $\nu_3$ $SO_2$ | 8 |
| 2926 | $3\nu_4$ $CD_4$ | 14 | 1312 | $\nu$(S=O) ASA | 9,12,13 |
| 2618 | $\nu$(OD) | 9 | 1242 | $\nu$(S=O) SA | 11 |
| 2534 | $\nu$(OD) | 9 | 1185 | $\nu$(S=O) ASA | 9,12,13 |
| 2440 | $\nu$(CD) | 9 | 1148 | $\nu_1$ $SO_2$ | 8 |
| 2392 | $\nu$(OD) | 9 | 1089 | $\delta$(SOD) ASA, SA | 9,11-13 |
| 2378 | $\nu$(CD) | 9 | 1049 | $\nu$(SO) | 9 |
| 2354 | $\nu$(CD) | 9 | 1001 | $\nu$(CO) | 9 |
| 2341 | $\nu$(CD) | 9 | 991 | $\nu_4$ $CD_4$ | 14 |
| 2337 | $\nu_3$ $CO_2$ | 10 | 940 | $\rho$(CH) ASA, $\nu$(SO) SA | 9,11-13 |
| 2318 | $\nu$(CD) | 9 | 568 | $\delta(SO_2)$ ASA | 9,12,13 |
| 2298 | $\nu$(CD) | 9 | 532 | $\nu_2$ $SO_2$ | 8 |
| 2274 | $\nu$(CD) | 9 | | | |
| 2250 | $\nu_3$ $CD_4$ | 14 | | | |
| 2236 | $\nu_{10}$ $C_2D_6$ | 16 | | | |
| 2213 | $\nu$(S-OD) | 9 | | | |
| 2212 | $\nu$(CD) | 9 | | | |
| 2137 | $\nu_1$ (CO) | 10 | | | |
| 2130 | $\nu$(C≡C) | 9 | | | |
| 2129 | $\nu$(OD) | 9 | | | |
| 2118 | $\nu$(C≡C) | 9 | | | |
| 2090 | $\nu$(C≡C) | 9 | | | |
| 2078 | $\nu_5$ $C_2D_6$ | 16 | | | |
| 2070 | $\nu_2 + \nu_4$ $CD_4$ | 14 | | | |
| 2035 | $\nu_1$ (OCS) | 1 | | | |
| 1977 | $\nu$(C≡C) | 9 | | | |
| 1854 | $\nu_3 + \nu_6$ $C_2D_6$ | 16 | | | |
| 1675 | $\nu$(C=O) | 9 | | | |
| 1596 | $\nu$(C=O) | 9 | | | |
| 1516 | $\nu_3$ $(CS_2)$ | 1 | | | |

**Supplementary Table 7.** Infrared absorption assignments for $SO_2/CD_4$.

| Residue (320 K) | | |
|---|---|---|
| Position ($cm^{-1}$) | Assignment | References |
| 2221 | $\nu$(OD) | 9 |
| 2068 | $\nu$(OD) | 9 |
| 1958 | $\nu$(C≡C) | 9 |
| 1831 | $\nu$(C≡C) | 9 |
| 1710 | $\nu$(C=O) | 9 |
| 1465 | $\delta(CD_3)$ ASA | 9,12,13 |
| 1314 | $\nu$(S=O) ASA | 9,12,13 |
| 1182 | $\nu$(S=O) ASA, SA | 9,11-13 |
| 991 | ν(S–OD) ASA, SA | 9,11-13 |
| 911 | $\nu$(CO) | 9 |
| 570 | $\delta(SO_2)$ ASA | 9,12,13 |

**Supplementary Table 8.** Infrared absorption assignments for $SO_2/H_2O/CH_4$ ices before irradiation.

| Peaks before irradiation (10 K) | | |
|---|---|---|
| Position ($cm^{-1}$) | Identity | References |
| 4302 | $\nu_3+\nu_4$ $CH_4$ | 2 |
| 4203 | $\nu_1+\nu_4$ $CH_4$ | 2 |
| 3662 | OH Dangling $H_2O$ | 17 |
| 3591 | $\nu_1$(O) $H_2O$ | 17 |
| 3446 | $\nu_3$(L) $H_2O$ | 17 |
| 3301 | $\nu_3$(L) $H_2O$ | 17 |
| 3218 | $\nu_3$(T) $H_2O$ | 17 |
| 3142 | $\nu_1$(T) $H_2O$ | 17 |
| 3008 | $\nu_3$ $CH_4$ | 2 |
| 3001 | $\nu_1$(I) $H_2O$ | 17 |
| 2902 | $\nu_1$ $CH_4$ | 2 |
| 2818 | $\nu_2+\nu_4$ $CH_4$ | 2 |
| 1659 | $\nu_2$ $H_2O$ | 17 |
| 1327 | $\nu_3$ $SO_2$ | 8 |
| 1302 | $\nu_4$ $CH_4$ | 2 |
| 1154 | $\nu_1$ $SO_2$ | 8 |
| 600-960 | $\nu_L$ $H_2O$ | 17 |
| 527 | $\nu_2$ $SO_2$ | 8 |

**Supplementary Table 9.** Infrared absorption assignments for $SO_2/H_2O/CH_4$ experiments.

| Peaks After Irradiation (10 K) | | | | | | | |
|---|---|---|---|---|---|---|---|
| Position ($cm^{-1}$) | Assignment | Dose (nA) | References | Position ($cm^{-1}$) | Assignment | Dose (nA) | References |
| 4302 | $\nu_3 + \nu_4$ $CH_4$ | 100, 1000 | 2 | 1185 | $\nu$(S=O) ASA, SA | 100, 1000, 5000 | 9,11-13 |
| 4203 | $\nu_1 + \nu_4$ $CH_4$ | 100, 1000 | 2 | 1152 | $\nu_1$ $SO_2$ | 100, 1000 | 8 |
| 3593, 3603 | OH Dangling | 100, 1000 | 17 | 1109 | $\delta$(SOH) ASA | 5000 | 9,12,13 |
| 3491, 3497, [illegible] | $\nu_1$(O) $H_2O$ | 100, 1000, 5000 | 17 | 1051 | $\nu$(SO) | 1000, 5000 | 9 |
| 3406, 3408, [illegible] | $\nu_3$(L) $H_2O$ | 100, 1000, 5000 | 17 | 1036 | $\nu$(CO) | 100 | 9 |
| 3299 | $\nu_3$(L) $H_2O$ | 5000 | 17 | 1016, 1019 | $\nu$(CO) | 1000, 5000 | 9 |
| 3230, 3224 | $\nu_3$(T) $H_2O$ | 100, 1000 | 17 | 868, 878, 877 | $\nu_L$ $H_2O$ | 100, 1000, 5000 | 17 |
| 3135, 3125, [illegible] | $\nu_1$(T) $H_2O$ | 100, 1000, 5000 | 17 | 790, 790, 779 | $\nu_L$ $H_2O$ | 100, 1000, 5000 | 17 |
| 3125 | $\nu$(OH) | 1000 | 9 | 668 | $\nu_2$ ($CO_2$) | 5000 | 10 |
| 3008 | $\nu_3$ $CH_4$ | 100, 1000, 5000 | 2 | 652 | $\nu_L$ $H_2O$ | 5000 | 17 |
| 2996 | $\nu$(SO-H) ASA | 100, 5000 | 9,12,13 | | | | |
| 2987 | $\nu$(OH) | 1000 | 9 | | | | |
| 2976 | $\nu_{10}$ $C_2H_6$ | 1000 | 2 | | | | |
| 2938 | $\nu_8 + \nu_{11}$ $C_2H_6$ | 1000 | 2 | | | | |
| 2903 | $\nu$(CH) | 100, 1000 | 9 | | | | |
| 2882 | $\nu_5$ $C_2H_6$ | 1000 | 2 | | | | |
| 2831 | $\nu$(CH) | 1000 | 9 | | | | |
| 2817 | $\nu$(OH) acid | 100 | 9,11 | | | | |
| 2815 | $\nu$(CH) | 1000 | 9 | | | | |
| 2497, 2565 | $\nu$(SO-H) ASA | 1000, 5000 | 9,12,13 | | | | |
| 2342 | $\nu_3$ ($CO_2$) | 1000, 5000 | 10 | | | | |
| 2203 | $\nu$(OH) acid | 5000 | 9,11 | | | | |
| 2189 | $\nu_2 + \nu_4$ ($CH_4$) | 1000 | 2 | | | | |
| 2137 | $\nu_1$ CO | 1000, 5000 | 10 | | | | |
| 1715 | $\nu$(CO) | 5000 | 9 | | | | |
| 1676 | $\nu_2$ $H_2O$ | 100, 1000, 5000 | 17 | | | | |
| 1606 | $\nu$(C=O) | 5000 | 9 | | | | |
| 1486 | $\delta$(CH) | 5000 | 9 | | | | |
| 1439 | $\delta$(CH) ASA | 5000 | 9,12,13 | | | | |
| 1329 | $\nu_3$ $SO_2$ | 100, 1000, 5000 | 8 | | | | |
| 1303 | $\nu_4$ $CH_4$ | 100, 1000, 5000 | 2 | | | | |
| 1274 | $\delta$(OH) SA | 100 | 11 | | | | |

**Supplementary Table 10.** Infrared absorption assignments for $SO_2/H_2O/CH_4$ experiments.

| Residue (320 K) | | | |
|---|---|---|---|
| Position ($cm^{-1}$) | Assignment | Dose (nA) | References |
| 3394, 3248 | *v*(OH) | 1000, 5000 | 9 |
| 3217, 3111 | *v*(OH) | 1000, 5000 | 9 |
| 2832 | *v*(SO-H) ASA | 5000 | 9,12,13 |
| 2528 | *v*(SO-H) ASA | 5000 | 9,12,13 |
| 2166 | *v*(OH) acid | 5000 | 9,11 |
| 1459, 1440 | *δ*(CH) ASA | 100, 1000, 5000 | 9,12,13 |
| 1320 | *v*(S=O) ASA | 100 | 9,12,13 |
| 1270, 1282 | *δ*(OH) SA | 100, 1000, 5000 | 9 |
| 1200 | *v*(S=O) ASA, SA | 100, 1000, 5000 | 9,11-13 |
| 1125 | *δ*(SOH) ASA | 5000 | 9,12,13 |
| 1036 | *v*(CO) | 5000 | 9 |
| 963, 938 | *ρ*(CH) ASA, *v*(SO) SA | 100, 1000 | 9,11-13 |
| 853 | *v*(SO) ASA | 5000 | 9,12,13 |
| 798 | *v*(CS) ASA | 1000 | 9,12,13 |

**Supplementary Table 11.** Identified alkylsulfonic acids as *tert*-butyldimethylsilyl derivatives by GC×GC–TOF-MS in the $SO_2/H_2O/CH_4$ and $SO_2/CH_4$ ice mixtures along with concentration estimates.

| Compound | $R_{t1}$[a] (min) | $R_{t2}$[b] (sec) | $[M]^{+\bullet}$ | $[M-57]^{+\bullet}$ | $SO_2/H_2O/CH_4$ (nmol) | | | $SO_2/CH_4$ (nmol) | | |
|---|---|---|---|---|---|---|---|---|---|---|
| | | | | | Low Dose | Medium Dose | High Dose | Low Dose | Medium Dose | High Dose |
| Methylsulfonic acid $CH_3SO_2(OH)$ | 17.17 | 1.48 | 210 | 153 | $10^{-1}$ | $10^{-1}$ | $10^{-1}$ | $5\times10^{-1}$ | $5\times10^{-1}$ | $5\times10^{1}$ |
| Ethylsulfonic acid $CH_3CH_2SO_2(OH)$ | 19.00 | 1.23 | 224 | 167 | $5\times10^{-2}$ | $5\times10^{-2}$ | $5\times10^{-2}$ | $5\times10^{-2}$ | $5\times10^{-2}$ | $5\times10^{-1}$ |
| 1-Propylsulfonic acid $CH_3CH_2SO_2(OH)$ | 20.92 | 1.19 | 238 | 181 | n.d. | n.d. | $10^{-3}$ | n.d. | $10^{-3}$ | $5\times10^{-2}$ |
| Sulfuric Acid ($H_2SO_4$) | 25.50 | 0.8 | n.d. | 269 | n.d. | n.d. | $10^{-1}$ | n.d. | $10^{-1}$ | $10^{1}$ |

Note. n.d. is not detected
[a] GC×GC retention time 1st dimension.
[b] GC×GC retention time 2nd dimension.

**Supplementary Table 12.** Optical constants for small molecules tracked during irradiation and TPD in the infrared spectra.

| Molecule | Position ($cm^{-1}$) | A (cm $molecule^{-1}$) | Reference |
|---|---|---|---|
| $CH_4$ | 1303 | $9.71 \times 10^{-18}$ | 18 |
| $SO_2$ | 1333 | $1.47 \times 10^{-17}$ | 19 |
| $C_2H_6$ | 2976 | $2.2 \times 10^{-17}$ | 20 |
| $CH_3$ | 3160 | $2.88 \times 10^{-18}$ | 21 |
| $SO_3$ | 1385 | $3.0 \times 10^{-17}$ | 22 |
| MSA | 1200 | $5.0 \times 10^{-17}$ | estimated |

MSA: methylsulfonic acid

**Supplementary Table 13.** Change in column density before and after irradiation of the $SO_2/CH_4$ ices.

| | Change in column density (molecule $cm^{-2}$) | | |
|---|---|---|---|
| Identity | Low Dose | Medium Dose | High Dose |
| $CH_4$ | $-6.1 \times 10^{16}$ | $-7.4 \times 10^{16}$ | $-1.5 \times 10^{17}$ |
| $SO_2$ | $-2.9 \times 10^{16}$ | $-1.3 \times 10^{17}$ | $-1.6 \times 10^{17}$ |
| $C_2H_6$ | $2.6 \times 10^{15}$ | $1.3 \times 10^{16}$ | $4.3 \times 10^{15}$ |
| $SO_3$ | $3.4 \times 10^{15}$ | 0.0 | $7.0 \times 10^{15}$ |
| $CH_3$ | 0.0 | n.d. | n.d. |

Note n.d. is not detected.

**Supplementary Table 14.** Rate constants derived from iterative fitting of reaction scheme.

| Reaction | | Dose | | | Units |
|---|---|---|---|---|---|
| | | 100 nA | 1000 nA | 5000 nA | |
| $SO_2$ | → $SO + O(^3P/^1D)$ | $5.7 \pm 1.3 \times 10^{-6}$ | $6.8 \pm 1.0 \times 10^{-6}$ | $5.3 \pm 1.1 \times 10^{-5}$ | $s^{-1}$ |
| $SO_2 + O(^3P/^1D)$ | → $SO_3$ | $8.2 \pm 2.5 \times 10^{-22}$ | $2.0 \pm 0.7 \times 10^{-22}$ | $3.65 \pm 0.05 \times 10^{-22}$ | $cm^2$ $molecule^{-1}$ $s^{-1}$ |
| $[SO_2]_2$ | → $SO + SO_3$ | $1.3 \pm 0.1 \times 10^{-22}$ | $7.0 \pm 0.2 \times 10^{-22}$ | $4.0 \pm 0.3 \times 10^{-22}$ | $cm^2$ $molecule^{-1}$ $s^{-1}$ |
| $CH_4$ | → $CH_3 + H(^2S)$ | $1.0 \pm 0.5 \times 10^{-5}$ | $2.3 \pm 0.3 \times 10^{-4}$ | $9.5 \pm 0.3 \times 10^{-4}$ | $s^{-1}$ |
| $[CH_4]_2$ | → $CH_3 + CH_3 + 2H/H_2$ | $1.27 \pm 0.05 \times 10^{-23}$ | $8.6 \pm 2.1 \times 10^{-22}$ | $8.25 \pm 0.07 \times 10^{-22}$ | $cm^2$ $molecule^{-1}$ $s^{-1}$ |
| $SO_3 + CH_3$ | → $CH_3SO_3$ | $7.2 \pm 0.8 \times 10^{-20}$ | $7.2 \pm 0.5 \times 10^{-19}$ | $6.0 \pm 1.5 \times 10^{-18}$ | $cm^2$ $molecule^{-1}$ $s^{-1}$ |
| $CH_3SO_3 + H$ | → $CH_3SO_2(OH)$ | $1.2 \pm 0.4 \times 10^{-19}$ | $7.5 \pm 3.4 \times 10^{-19}$ | $9.0 \pm 1.4 \times 10^{-18}$ | $cm^2$ $molecule^{-1}$ $s^{-1}$ |
| $CH_3 + CH_3$ | → $C_2H_6$ | $8.1 \pm 0.2 \times 10^{-19}$ | $5.6 \pm 0.8 \times 10^{-20}$ | $2.8 \pm 0.3 \times 10^{-17}$ | $cm^2$ $molecule^{-1}$ $s^{-1}$ |
| $CH_3 + H(^2S)$ | → $CH_4$ | - | $7.9 \pm 0.5 \times 10^{-20}$ | $9.0 \pm 3.5 \times 10^{-18}$ | $cm^2$ $molecule^{-1}$ $s^{-1}$ |
| $SO + O(^3P/^1D)$ | → $SO_2$ | - | $2.5 \pm 0.2 \times 10^{-20}$ | $2.5 \pm 0.2 \times 10^{-20}$ | $cm^2$ $molecule^{-1}$ $s^{-1}$ |
| $CH_3SO_2(OH)$ | → Products | - | $4.3 \pm 0.6 \times 10^{-20}$ | $3.0 \pm 0.2 \times 10^{-4}$ | $cm^2$ $molecule^{-1}$ $s^{-1}$ |
| $CH_3$ | → Products | $5.9 \pm 1.1 \times 10^{-3}$ | $5.9 \pm 1.1 \times 10^{-3}$ | $5.9 \pm 1.1 \times 10^{-3}$ | $s^{-1}$ |
| $SO_3$ | → Products | $8.7 \pm 0.3 \times 10^{-4}$ | $9.5 \pm 2.0 \times 10^{-3}$ | $3.0 \pm 1.0 \times 10^{-3}$ | $s^{-1}$ |
| $[SO_2]_2$ | → Products | - | - | $2.65 \pm 0.08 \times 10^{-21}$ | $cm^2$ $molecule^{-1}$ $s^{-1}$ |
| $[CH_4]_2$ | → Products | - | - | $1.7 \pm 0.4 \times 10^{-21}$ | $cm^2$ $molecule^{-1}$ $s^{-1}$ |
| $C_2H_6 + SO$ | → Products | - | - | $2.5 \pm 0.3 \times 10^{-18}$ | $cm^2$ $molecule^{-1}$ $s^{-1}$ |
| $SO$ | → Products | - | - | $2.0 \pm 1.0 \times 10^{-3}$ | $s^{-1}$ |

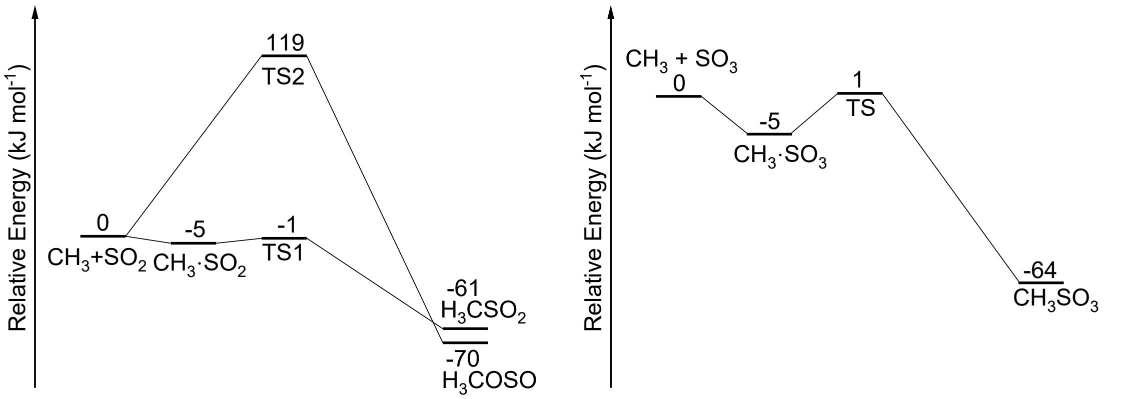


**Supplementary Figure 8. Calculated potential energy surfaces for $SO_2 + CH_3$ and $SO_3 + CH_3$.** Radical addition of $CH_3^{\bullet}$ to $SO_2$ computed at the CCSD(T)/aug-cc-pV(Q+d)Z//CCSD(T)/6-311++G(2df, p) level (left) calculated by Ratliff et al.[23] and $CH_3$ addition to $SO_3$ computed at the G3XMP2//B3LYP/6-311+G(3df,2p) level of theory by Cao et al[7].

**Supplementary References**


1 Maity, S. & Kaiser, R. I. Electron irradiation of carbon disulfide–oxygen ices: Toward the formation of sulfur-bearing molecules in interstellar ices. *Astrophys. J.* **773**, 184, (2013).

2 Bennett, C. J., Jamieson, C. S., Osamura, Y. & Kaiser, R. I. Laboratory studies on the irradiation of methane in interstellar, cometary, and solar system ices. *Astrophys. J.* **653**, 792, (2006).

3 Gerakines, P. A., Schutte, W. A. & Ehrenfreund, P. Ultraviolet processing of interstellar ice analogs. I. Pure ices. *Astron. Astrophys.* **312**, 289–305, (1996).

4 Hepp, M. & Herman, M. Weak combination bands in the 3-μm region of ethane. *I. Mol. Spectrosc.* **197**, 56-63, (1999).

5 Turner, A. M., Abplanalp, M. J., Blair, T. J., Dayuha, R. & Kaiser, R. I. An Infrared Spectroscopic Study Toward the Formation of Alkylphosphonic Acids and Their Precursors in Extraterrestrial Environments. *Astrophys. J. Suppl.* **234**, 6, (2018).

6 Turner, A. M. *et al.* A photoionization mass spectroscopic study on the formation of phosphanes in low temperature phosphine ices. *Phys. Chem. Chem. Phys.* **17**, 27281–27291, (2015).

7 Cao Jia, W. W.-l., Gao Lou-jun, Fu feng. Mechanism and Thermodynamic Properties of $CH_3SO_3$ Decomposition. *Acta Physico-Chimica Sinica* **29**, 1161–1167, (2013).

8 Moore, M. H. Studies of proton-irradiated $SO_2$ at low temperatures: Implications for lo. *Icarus* **59**, 114–128, (1984).

9 Socrates, G. *Infrared and Raman Characteristic Group Frequencies*. 3rd edn, (2004).

10 Gerakines, P. A., Schutte, W. A., Greenberg, J. M. & van Dishoeck, E. F. The infrared band strengths of $H_2O$, CO and $CO_2$ in laboratory simulations of astrophysical ice mixtures. *Astron. Astrophys.* **296**, 810, (1995).

11 Zhong, L. & Parker, S. F. Structure and vibrational spectroscopy of methanesulfonic acid. *Royal Soc. Open Sci.* **5**, 181363, (2018).

12 Durig, J. R., Zhou, L., Schwartz, T. & Gounev, T. Fourier transform Raman spectrum, vibrational assignment and ab initio calculation of methanesulfonic acid in the gas and liquid phases. *J. Raman Spectrosc.* **31**, 193–202, (2000).

13 Nash, K. L., Sully, K. J. & Horn, A. B. Observations on the interpretation and analysis of sulfuric acid hydrate infrared spectra. *J. Phys. Chem. A* **105**, 9422–9426, (2001).

14 Calvani, P., Lupi, S. & Maselli, P. The infrared spectrum of solid $CD_4$. *J. Chem. Phys.* **91**, 6737–6742, (1989).

15 He, J., Gao, K., Vidali, G., Bennett, C. J. & Kaiser, R. I. Formation of molecular hydrogen from methane ice. *Astrophys. J.* **721**, 1656, (2010).

16 Abplanalp, M. J. & Kaiser, R. I. Complex hydrocarbon chemistry in interstellar and solar system ices revealed: A combined infrared spectroscopy and reflectron time-of-flight mass spectrometry analysis of ethane ($C_2H_6$) and d6-ethane ($C_2D_6$) ices exposed to ionizing radiation. *Astrophys. J.* **827**, 132, (2016).

17 Hagen, W., Tielens, A. & Greenberg, J. The infrared spectra of amorphous solid water and ice Ic between 10 and 140 K. *Chem. Phys.* **56**, 367–379, (1981).

18 Gerakines, P. A. & Hudson, R. L. Infrared spectra and optical constants of elusive amorphous methane. *Astrophys. J. Lett.* **805**, L20, (2015).

19 Garozzo, M., Fulvio, D., Gomis, O., Palumbo, M. E. & Strazzulla, G. H-implantation in $SO_2$ and $CO_2$ ices. *Planet. Space Sci.* **56**, 1300–1308, (2008).

20 Hudson, R. L., Gerakines, P. A. & Moore, M. H. Infrared spectra and optical constants of astronomical ices: II. Ethane and ethylene. *Icarus* **243**, 148–157, (2014).

21 Snelson, A. Infrared matrix isolation spectrum of the methyl radical produced by pyrolysis of methyl iodide and dimethyl mercury. *J. Phys. Chem.* **74**, 537–544, (1970).

22 Garozzo, M., Fulvio, D., Kanuchova, Z., Palumbo, M. E. & Strazzulla, G. The fate of S-bearing species after ion irradiation of interstellar icy grain mantles. *A&A* **509**, A67, (2010).

23 Ratliff, B. J., Tang, X., Butler, L. J., Szpunar, D. E. & Lau, K.-C. Determining the $CH_3SO_2 \rightarrow CH_3+SO_2$ barrier from methylsulfonyl chloride photodissociation at 193 nm using velocity map imaging. *J. Chem. Phys.* **131**, 044304, (2009).
